# Supplementary material for: An atlas of the C. elegans starvation response reveals that transcription is necessary to initiate transcriptional silencing in quiescent primordial germ cells
Source: bioRxiv. 2026 Sep 8:2026.09.04.749554. Preprint. [Version 1] doi: 10.64898/2026.09.04.749554 (PMC13622491; doi:10.64898/2026.09.04.749554)
Supplement: Supplementary file 1 [file NIHPP2026.09.04.749554V1-supplement-1.pdf]

bioRxiv preprint doi: https://doi.org/10.64898/2026.09.04.749554; this version posted September 9, 2026. The copyright holder for this preprint (which was not certified by peer review) is the author/funder, who has granted bioRxiv a license to display the preprint in perpetuity. It is made available under a CC-BY 4.0 International license.



## SUPPLEMENTARY MATERIALS

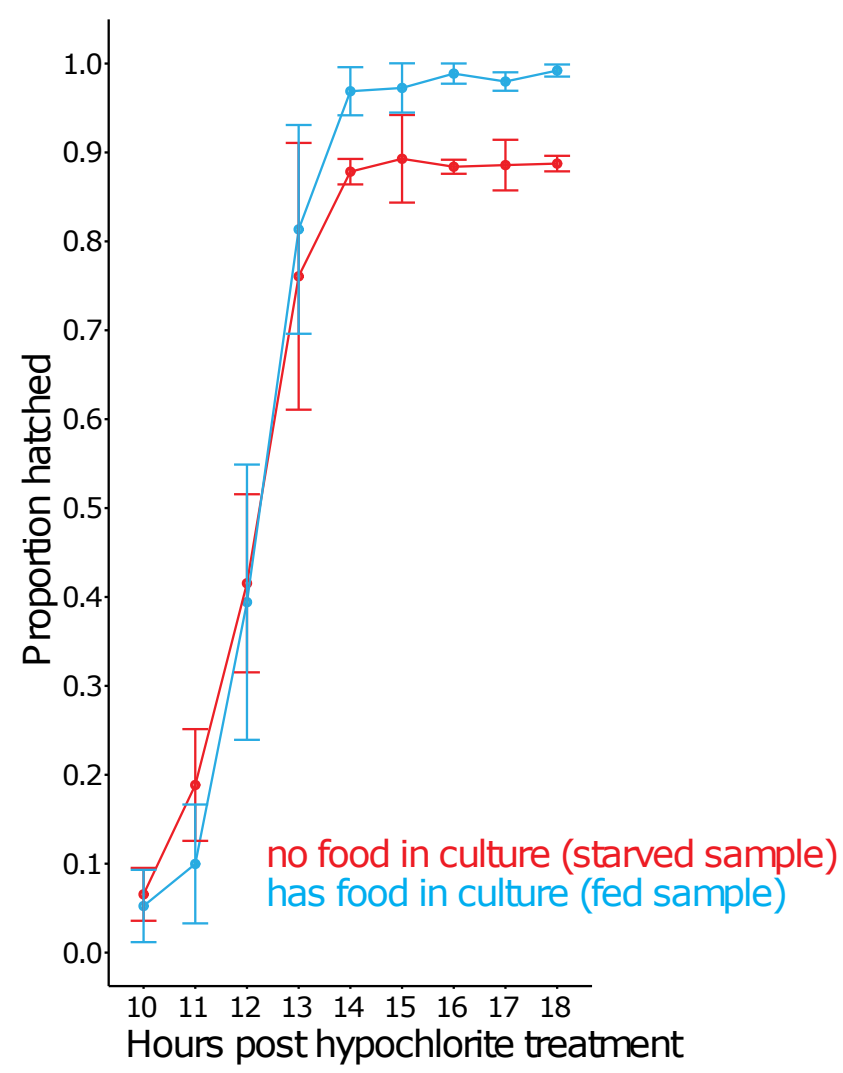


**Sup. Figure 1.** Proportion of hatched embryos (hatching efficiency) was measured

bioRxiv preprint doi: https://doi.org/10.64898/2026.09.04.749554; this version posted September 9, 2026. The copyright holder for this preprint (which was not certified by peer review) is the author/funder, who has granted bioRxiv a license to display the preprint in perpetuity. It is made available under a CC-BY 4.0 International license.



between 10 and 18 h after hypochlorite treatment (‘bleach’). Related to Fig. 1A. Three biological replicates were performed. Dots are average hatching efficiency across replicates. Error bars represent standard deviation (SD). At ~12 h after bleach, both starved and fed samples reached approximately 50% hatching. Therefore, 12 h after bleach was designated as the time point for hatching. Number of examined embryos (hatched and unhatched) is 96 +/- 15 (mean +/- SD).

bioRxiv preprint doi: https://doi.org/10.64898/2026.09.04.749554; this version posted September 9, 2026. The copyright holder for this preprint (which was not certified by peer review) is the author/funder, who has granted bioRxiv a license to display the preprint in perpetuity. It is made available under a CC-BY 4.0 International license.



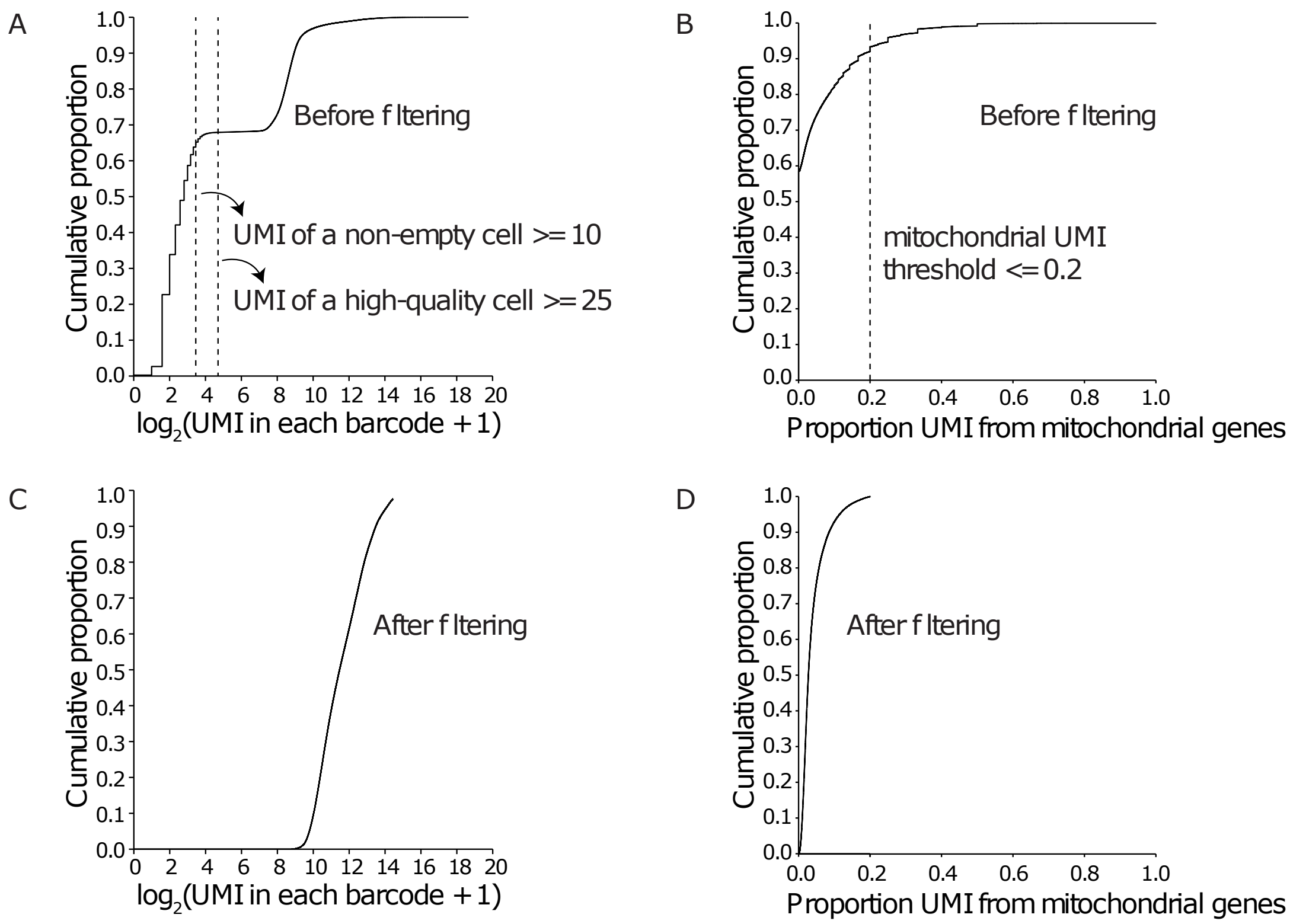


1443

1444 **Sup. Figure 2.** Quality control for single-cell RNA-seq samples. Related to Fig. 1. (A) Plot

1445 showing the cumulative distribution of $log_2$-transformed UMI counts in each barcode (cell)

bioRxiv preprint doi: https://doi.org/10.64898/2026.09.04.749554; this version posted September 9, 2026. The copyright holder for this preprint (which was not certified by peer review) is the author/funder, who has granted bioRxiv a license to display the preprint in perpetuity. It is made available under a CC-BY 4.0 International license.



before filtering. Filtering used at least 10 UMI and at least 25 UMI as the cutoff for non-empty cells and high-quality cells, respectively. (B) Plot showing the cumulative distribution of the proportion of UMI coming from mitochondrial genes in each barcode (cell) before filtering. Filtering used 0.2 as the cutoff for healthy cells (Taylor *et al*, 2021) (C) Plot showing the cumulative distribution of $\log_2$-transformed UMI counts in each barcode (cell) after filtering. (D) Plot showing the cumulative distribution of the proportion of UMI coming from mitochondrial genes in each barcode (cell) after filtering.

bioRxiv preprint doi: https://doi.org/10.64898/2026.09.04.749554; this version posted September 9, 2026. The copyright holder for this preprint (which was not certified by peer review) is the author/funder, who has granted bioRxiv a license to display the preprint in perpetuity. It is made available under a CC-BY 4.0 International license.



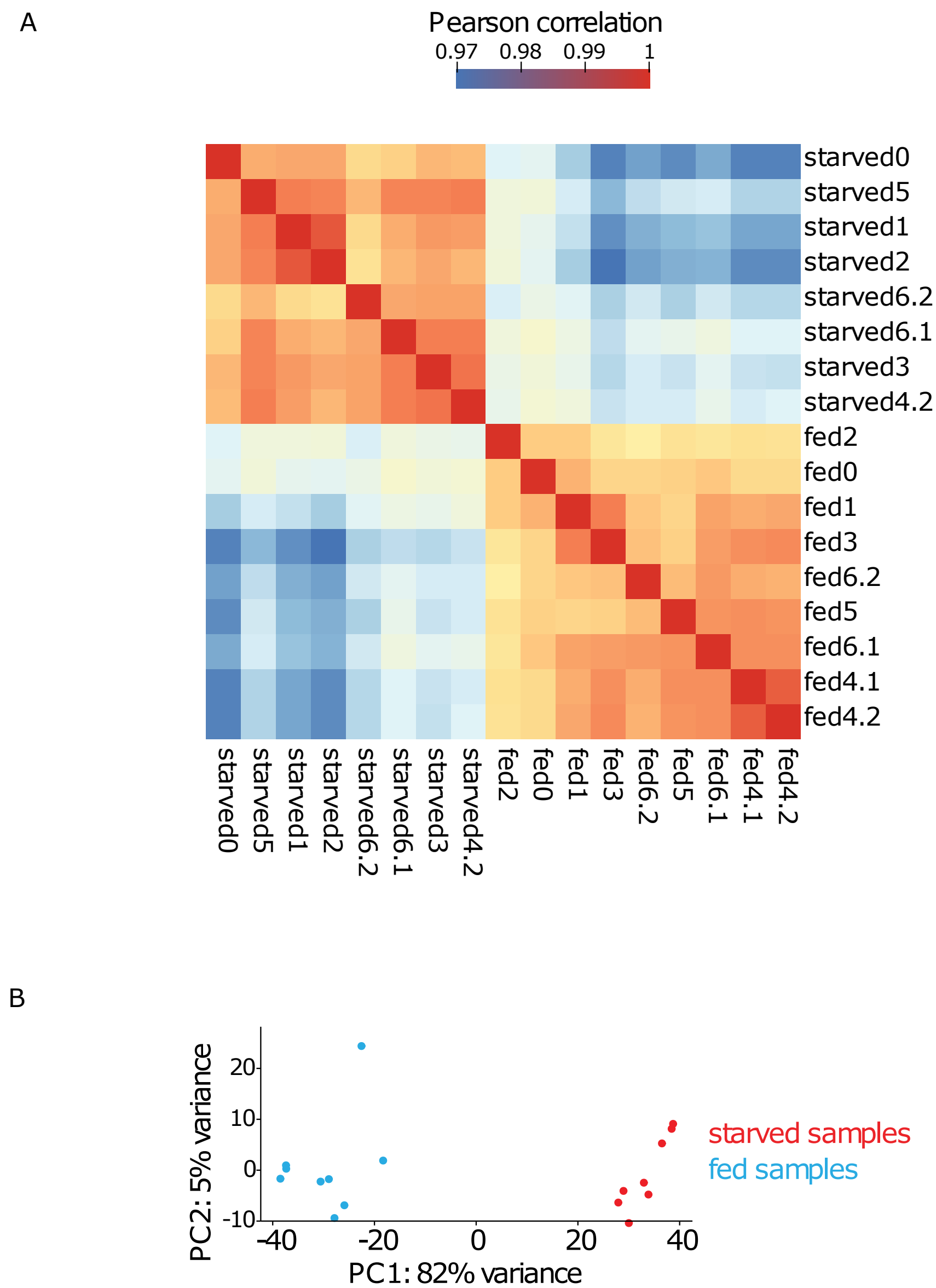


**Sup. Figure 3.** Single-cell RNA-seq reproducibly captured the starvation response. Related to Fig. 1 and 2. (A) Pearson correlation coefficient between each pair of samples (total: 9

bioRxiv preprint doi: https://doi.org/10.64898/2026.09.04.749554; this version posted September 9, 2026. The copyright holder for this preprint (which was not certified by peer review) is the author/funder, who has granted bioRxiv a license to display the preprint in perpetuity. It is made available under a CC-BY 4.0 International license.



fed samples and 8 starved samples) reveals reproducibility among fed and starved replicates as well as differences between fed and starved conditions. (B) Principal component analysis (PCA) of starved and fed pseudobulk samples. The first principal component is correlated with fed vs. starved and explains 82% of the variance, indicating a robust effect of nutrient availability on gene expression.

bioRxiv preprint doi: https://doi.org/10.64898/2026.09.04.749554; this version posted September 9, 2026. The copyright holder for this preprint (which was not certified by peer review) is the author/funder, who has granted bioRxiv a license to display the preprint in perpetuity. It is made available under a CC-BY 4.0 International license.



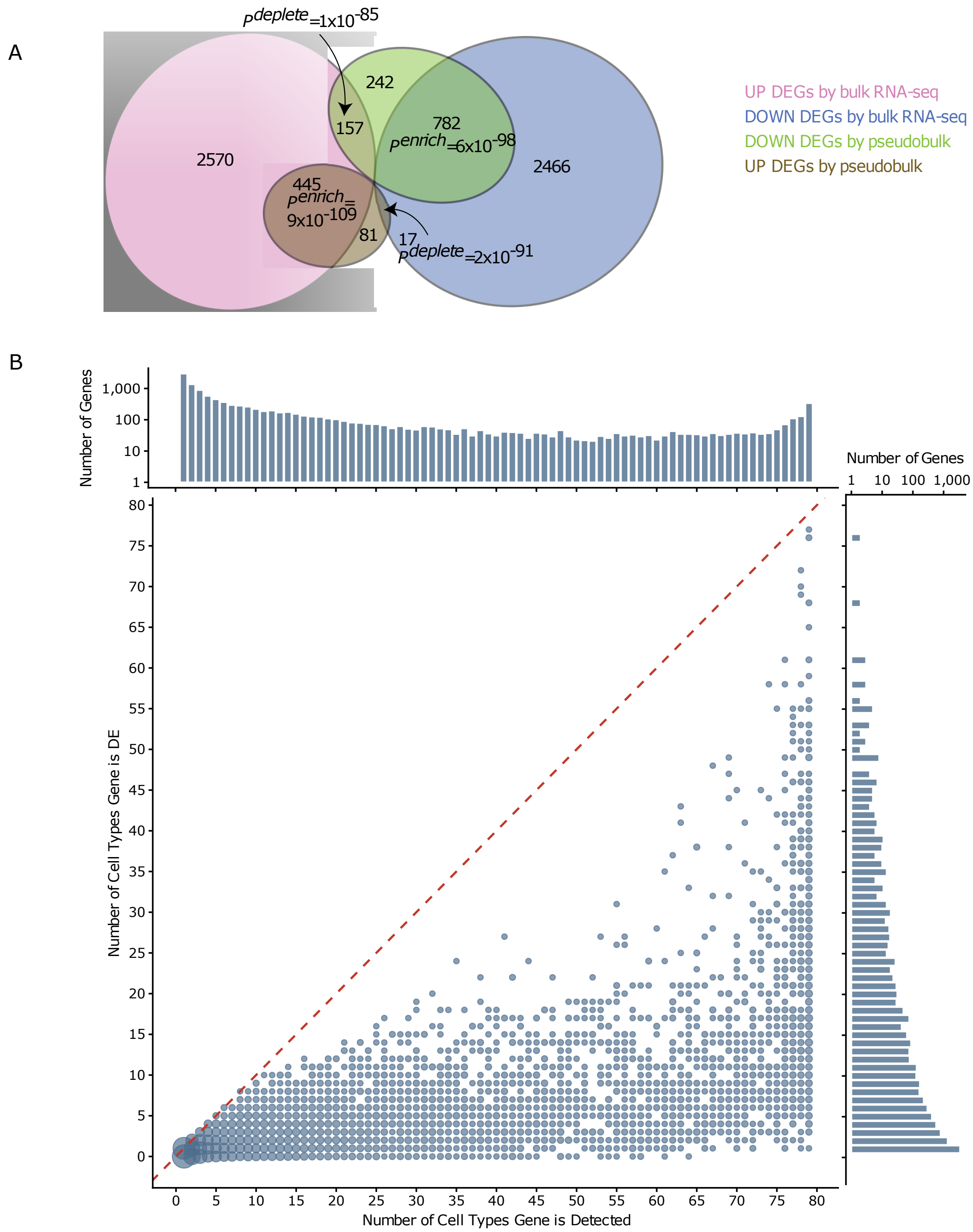


**Sup. Figure 4.** scRNA-seq data are corroborated by published bulk RNA-seq results and reveal that gene expression is detected more broadly than differential expression. Related

bioRxiv preprint doi: https://doi.org/10.64898/2026.09.04.749554; this version posted September 9, 2026. The copyright holder for this preprint (which was not certified by peer review) is the author/funder, who has granted bioRxiv a license to display the preprint in perpetuity. It is made available under a CC-BY 4.0 International license.



to Fig. 2. (A) Overlap among genes up and downregulated in starved vs. fed conditions in pseudobulk and a previous bulk mRNA-seq study (Webster *et al*, 2018). Fed and starved samples were collected 18 h and 24 h after bleach, with and without food, respectively, in the bulk mRNA-seq study, which are the same fed and starved time points as in our single-cell RNA-seq study. Hypergeometric tests were done to assess statistical significance of overlaps, with the background set being protein-coding genes detected in both scRNA-seq and bulk mRNA-seq. $P^{enrich}$ is enrichment p-value from the hypergeometric test. $P^{deplete}$ is 1 minus $P^{enrich}$. (B) Bubble plot relating distributions of the number of cell types genes are detected in and the number of cell types genes are differentially expressed in. A one-dimensional histogram is plotted separately for each axis. Dashed red line indicates y = x.

bioRxiv preprint doi: https://doi.org/10.64898/2026.09.04.749554; this version posted September 9, 2026. The copyright holder for this preprint (which was not certified by peer review) is the author/funder, who has granted bioRxiv a license to display the preprint in perpetuity. It is made available under a CC-BY 4.0 International license.



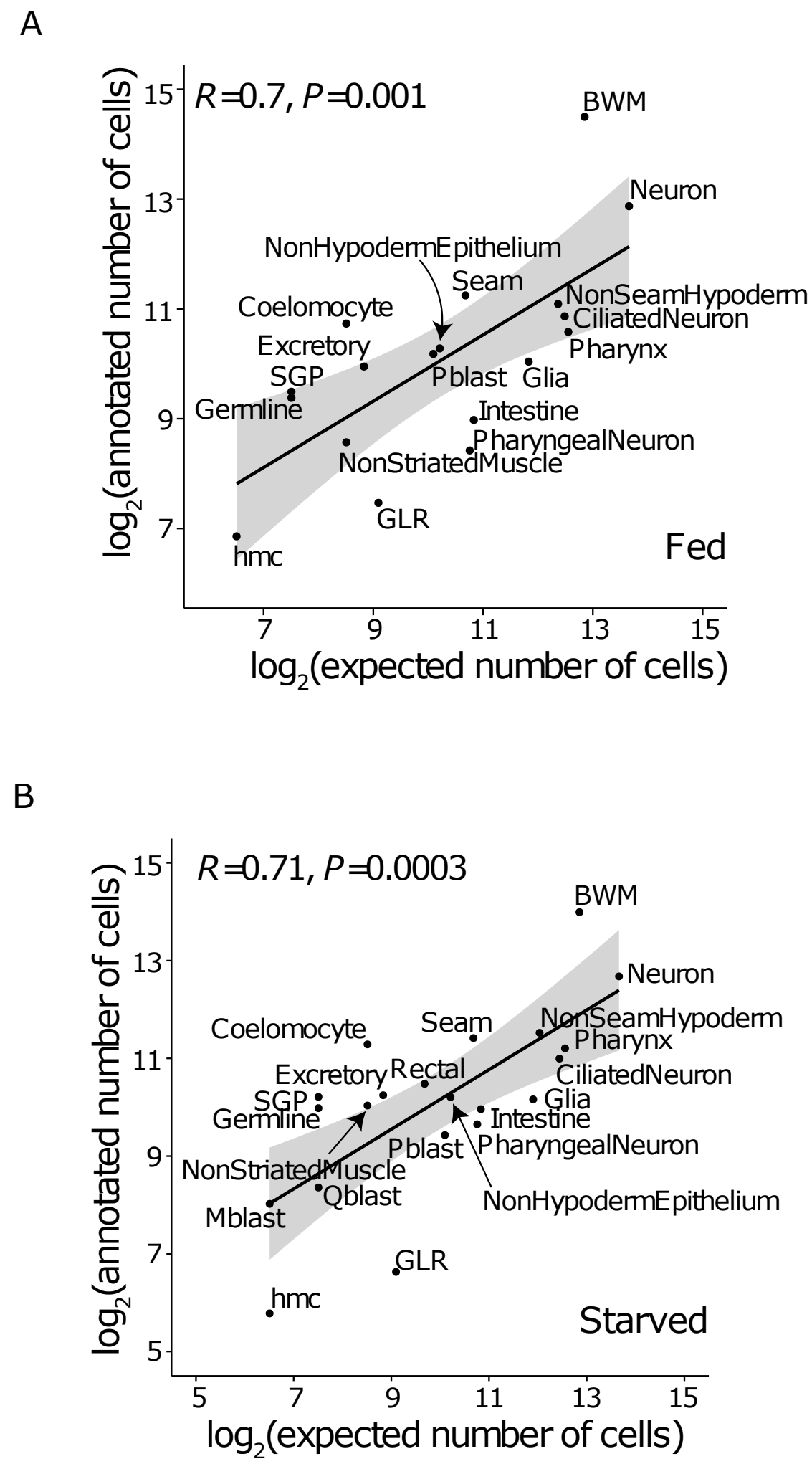


1477

1478 **Sup. Figure 5.** Single-cell RNA-seq reliably identified the major tissues. Related to Figure 3.

1479 (A, B) $Log_2$ transformed annotated vs. expected number of cells (based on the invariant

bioRxiv preprint doi: https://doi.org/10.64898/2026.09.04.749554; this version posted September 9, 2026. The copyright holder for this preprint (which was not certified by peer review) is the author/funder, who has granted bioRxiv a license to display the preprint in perpetuity. It is made available under a CC-BY 4.0 International license.



anatomy of young L1 larvae) for each tissue in fed (A) and starved (B) samples. Pearson correlation coefficients and p-values are indicated. The black line represents a linear regression, and the gray area represents a 95% confidence region. See Sup. Data 2 for a complete list of cell number per tissue in fed and starved conditions.

bioRxiv preprint doi: https://doi.org/10.64898/2026.09.04.749554; this version posted September 9, 2026. The copyright holder for this preprint (which was not certified by peer review) is the author/funder, who has granted bioRxiv a license to display the preprint in perpetuity. It is made available under a CC-BY 4.0 International license.



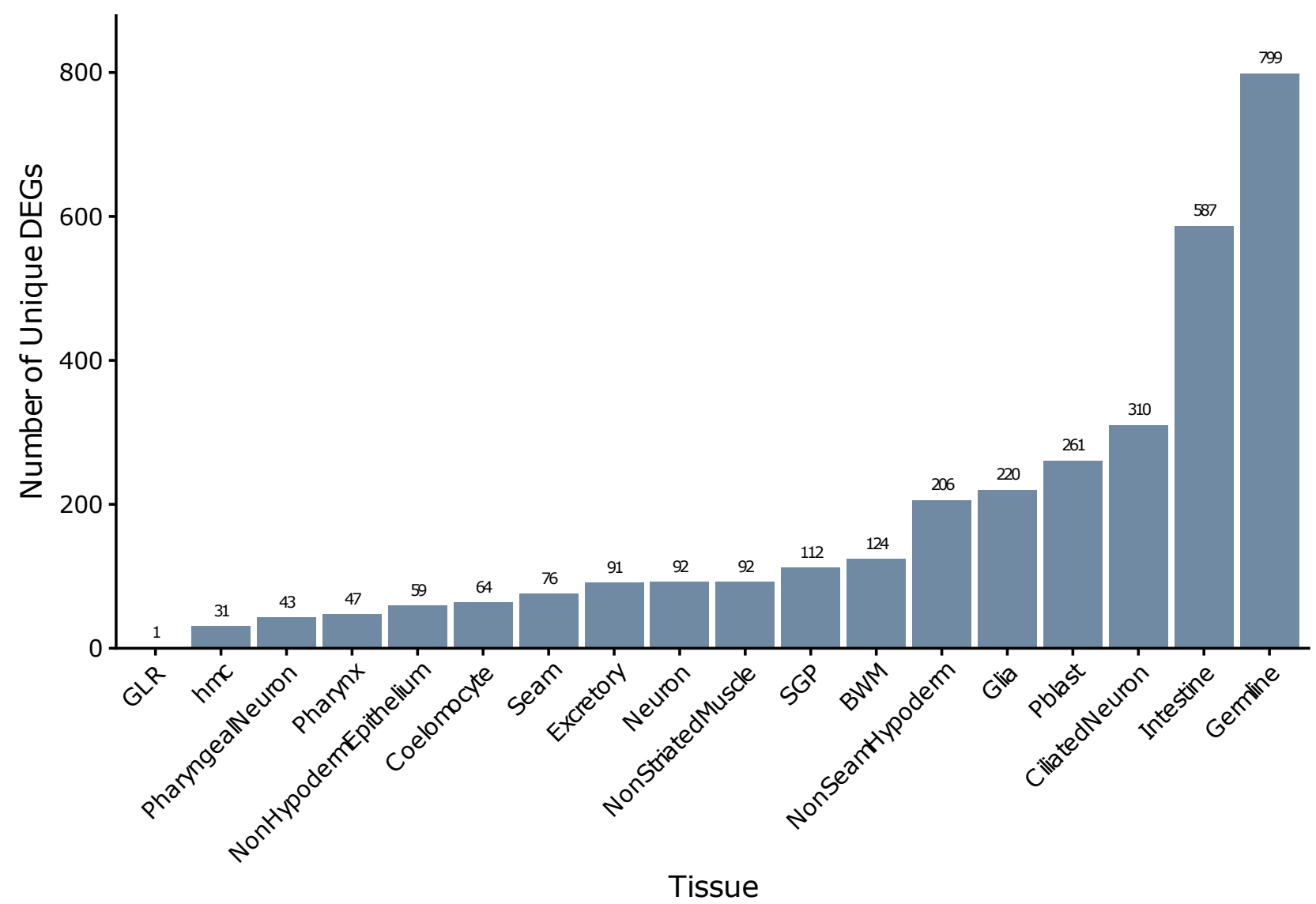


**Sup. Figure 6.** Many genes are differentially expressed in a single tissue. Related to Figure 4. The number of genes that are differentially expressed in a single tissue ('unique DEGs') is

bioRxiv preprint doi: https://doi.org/10.64898/2026.09.04.749554; this version posted September 9, 2026. The copyright holder for this preprint (which was not certified by peer review) is the author/funder, who has granted bioRxiv a license to display the preprint in perpetuity. It is made available under a CC-BY 4.0 International license.



plotted for each tissue. The germline and intestine are the sites of differential expression for the largest numbers of unique DEGs.

bioRxiv preprint doi: https://doi.org/10.64898/2026.09.04.749554; this version posted September 9, 2026. The copyright holder for this preprint (which was not certified by peer review) is the author/funder, who has granted bioRxiv a license to display the preprint in perpetuity. It is made available under a CC-BY 4.0 International license.



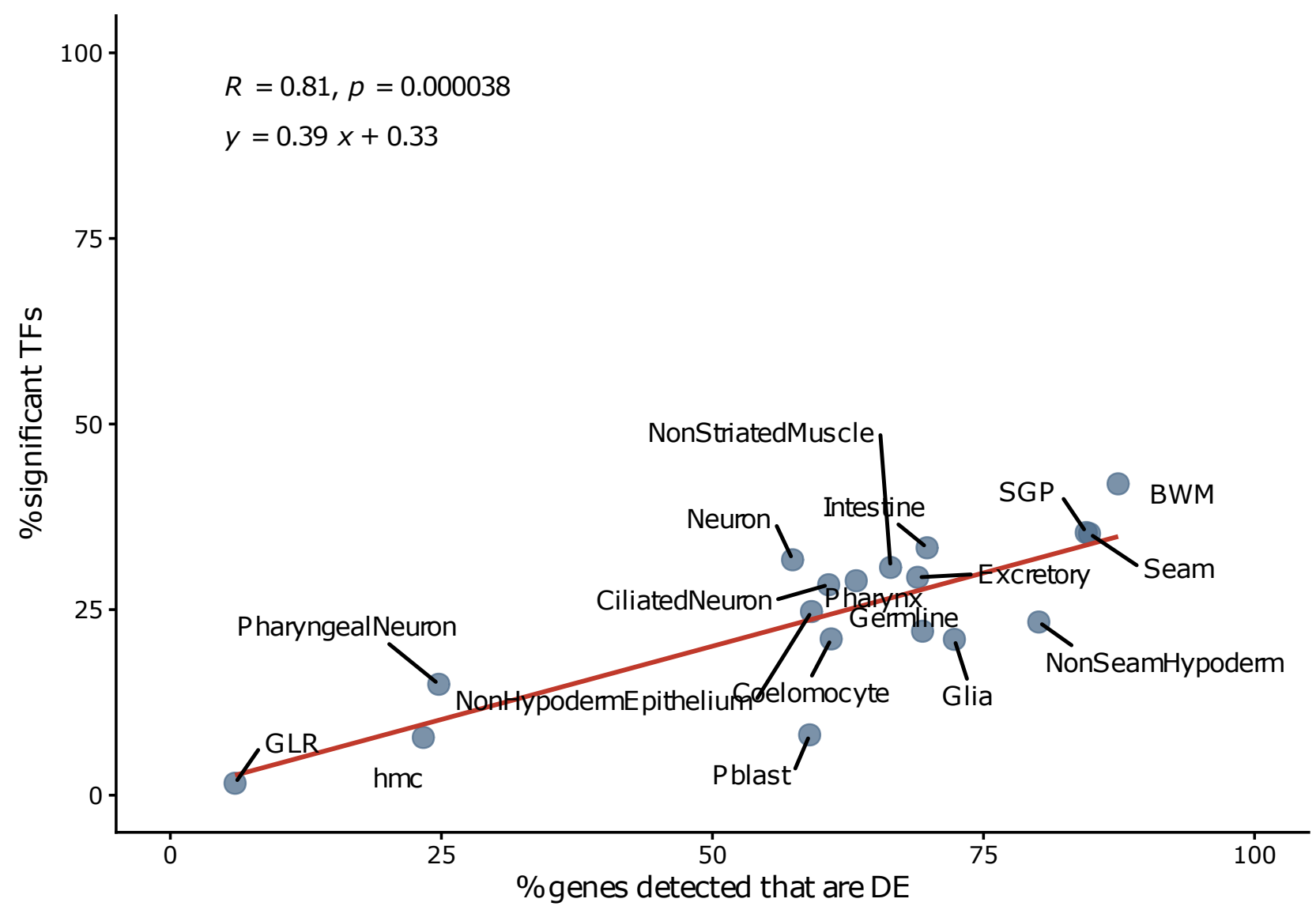


1491

1492 **Sup. Figure 7.** The proportion of TFs with significant tissue-specific differences in activity

1493 between fed and starved L1 larvae is proportional to the proportion of genes that

bioRxiv preprint doi: https://doi.org/10.64898/2026.09.04.749554; this version posted September 9, 2026. The copyright holder for this preprint (which was not certified by peer review) is the author/funder, who has granted bioRxiv a license to display the preprint in perpetuity. It is made available under a CC-BY 4.0 International license.



differentially expressed in that tissue. The percentage of genes detected in each tissue that are differentially expressed (DE) is plotted against the percentage of transcription factors (TFs) with significant activity estimates per tissue. The red line indicates the linear regression, and the Pearson correlation coefficient, p-value, and slope are given. Related to Figure 6.

bioRxiv preprint doi: https://doi.org/10.64898/2026.09.04.749554; this version posted September 9, 2026. The copyright holder for this preprint (which was not certified by peer review) is the author/funder, who has granted bioRxiv a license to display the preprint in perpetuity. It is made available under a CC-BY 4.0 International license.



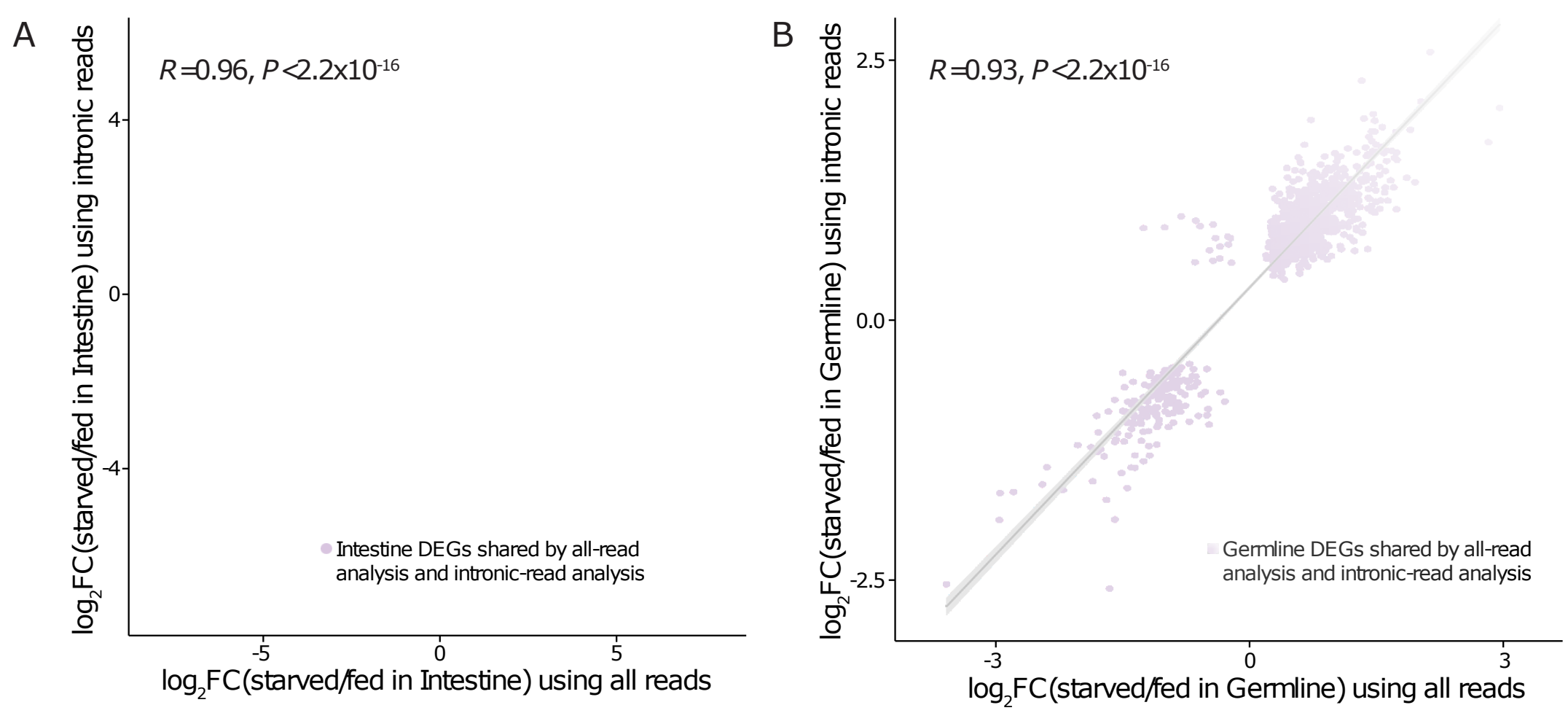


1500

1501 **Sup. Figure 8.** Comparison of differential expression (DE) analysis results using all reads

1502 and intronic reads. Related to Figure 7B-E. (A, B) $Log_2$fold-change ($log_2FC$) of differentially

bioRxiv preprint doi: https://doi.org/10.64898/2026.09.04.749554; this version posted September 9, 2026. The copyright holder for this preprint (which was not certified by peer review) is the author/funder, who has granted bioRxiv a license to display the preprint in perpetuity. It is made available under a CC-BY 4.0 International license.



expressed genes (DEGs) using all reads vs. intronic reads for the intestine (A) and germline (B). Pearson correlation coefficient and p-values are indicated. Only DEGs shared by intronic- and all-read analyses were analyzed. Black line represents a linear regression, and the very narrow gray area represents the 95% confidence region. See Sup. Data 4 and 8 for detailed differential expression analysis results using all reads and intronic reads, respectively.

bioRxiv preprint doi: https://doi.org/10.64898/2026.09.04.749554; this version posted September 9, 2026. The copyright holder for this preprint (which was not certified by peer review) is the author/funder, who has granted bioRxiv a license to display the preprint in perpetuity. It is made available under a CC-BY 4.0 International license.



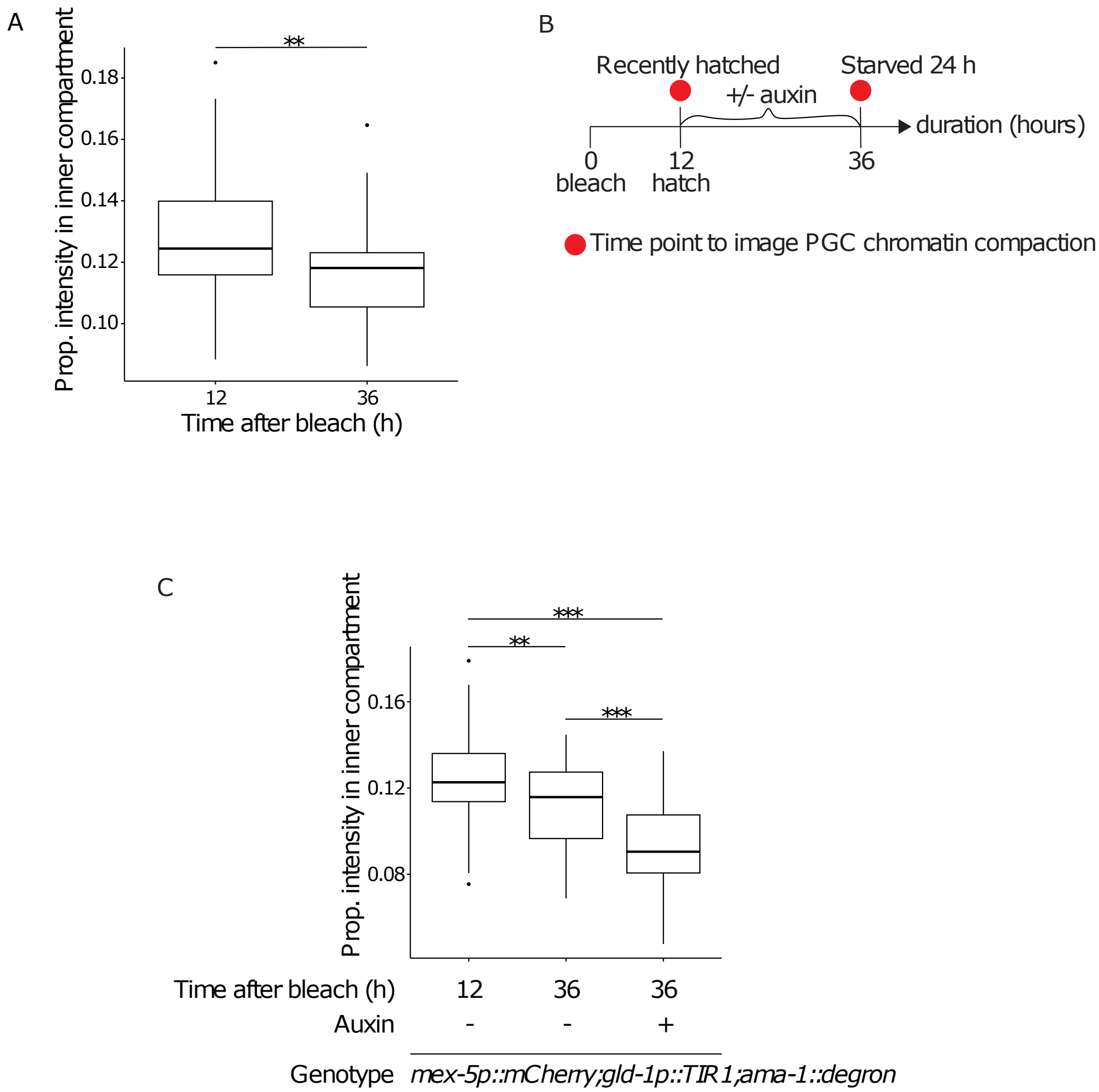


**Sup. Figure 9.** PGC chromatin hyper-compaction due to starvation and severe compaction due to degradation of the large subunit of RNAPII. (A) Proportion of PGC

bioRxiv preprint doi: https://doi.org/10.64898/2026.09.04.749554; this version posted September 9, 2026. The copyright holder for this preprint (which was not certified by peer review) is the author/funder, who has granted bioRxiv a license to display the preprint in perpetuity. It is made available under a CC-BY 4.0 International license.



chromatin intensity in the 'inner compartment' (11.1% as previously defined (Belew *et al*, 2021)) in recently hatched (12 h after bleach) vs 24 h starved (36 h after bleach) wild-type worms. Number of PGCs: 51 and 67 for recently hatched and 24 h starved worms, respectively. Because Bartlett's test rejected equal variance between the two conditions, an unpaired t-test was performed without pooling variances. (B) Schematic for the auxin-induced degradation (AID) experiment to degrade AMA-1/RNAPII and image PGC chromatin compaction. (C) Proportion of PGC chromatin intensity in the 'inner compartment' (11.1%) in recently hatched (12 h after bleach) vs 24 h starved (36 h after bleach) worms with or without auxin to degrade AMA-1/RNAPII. Number of PGCs: 57 for recently hatched worms, 40 for 24 h starved worms without auxin, and 37 for 24 h starved worms with auxin. Barlett's test did not reject equal variance among the three groups, so unpaired t-tests were performed with pooled variances. (A, C) **P<0.01; ***P<0.001.

bioRxiv preprint doi: https://doi.org/10.64898/2026.09.04.749554; this version posted September 9, 2026. The copyright holder for this preprint (which was not certified by peer review) is the author/funder, who has granted bioRxiv a license to display the preprint in perpetuity. It is made available under a CC-BY 4.0 International license.



| Probe_ID | Probe sequence (5' to 3') |
|---|---|
| 1 | cggccacctgtattaaaataat |
| 2 | ttttaaaagtcgaacctctgca |
| 3 | ttgtaatagtattctagaacca |
| 4 | ttctgaatttcagtttttttgt |
| 5 | aaattatttttctggtggggt |
| 6 | gggattttgcttcttaaaatat |
| 7 | ggttctcgatttctcgatgaaa |
| 8 | gtttttcgtacatttttactt |
| 9 | tttcatcttaactcatggaagt |
| 10 | cctgtctttttctatttttta |
| 11 | gtcggttaaaaaaaaattccca |
| 12 | aaaaaaaggtttcaggtgttgc |
| 13 | cagatttctcacttttcgtggt |
| 14 | gactacttttgcaacattcctt |
| 15 | ccaacatttgctgaaaactttc |
| 16 | gtgaactttcgtgtttatgtaa |
| 17 | aaaggcgcacttttcgtaattt |
| 18 | tgtgttttaattgttttacttt |
| 19 | gtcaagtgcgaaaaggcgtaaa |
| 20 | attttcactcgaagaatacggt |
| 21 | gtggaacagctgaaagtttttt |
| 22 | ctactccttctttgaaaaaaca |
| 23 | tgggggtttttgtgaaaaattt |
| 24 | aaattcgtttttttttactct |
| 25 | tcgtgttttcgatttttttttt |
| 26 | tctccaactacaaatcaatggt |
| 27 | tcctggtcctgcagaaaaaaaa |
| 28 | tttttactgatcgaaactgaga |
| 29 | tgaacgaactgttttttttca |
| 30 | gcgtcagtgatgtcaaaatttt |
| 31 | gtttcgttttaatgaaatgagc |
| 32 | tgttgacagctgaattaatatt |

**Sup. Table 1.** 5' to 3' sequence of 32 *aak-1/AMPK* intronic probes for smFISH. Related to Figure 6A-D.

bioRxiv preprint doi: https://doi.org/10.64898/2026.09.04.749554; this version posted September 9, 2026. The copyright holder for this preprint (which was not certified by peer review) is the author/funder, who has granted bioRxiv a license to display the preprint in perpetuity. It is made available under a CC-BY 4.0 International license.



**Sup. Data 1.** Post-quality-control sample metrics. UMI: Unique Molecular Identifier. Proportion (Prop.) genome was calculated by dividing number (No.) detected genes by the total number of genes in WS286 (46,926). Prop. protein-coding genome was calculated by dividing No. detected protein-coding genes by the total number of protein-coding genes in WS286 (19,983). Nine fed samples and eight starved samples passed quality control. Metrics for fed, starved, and total were calculated by aggregating all fed, all starved, and all samples, respectively. Related to Fig. 1.

**Sup. Data 2.** Information about marker genes used to annotate each cell type, how cell types were aggregated into tissues, cell type and tissue abbreviation rules, and the expected number of cells in each tissue in fed and starved conditions. Related to Figs. 1, 3, and S5.

**Sup. Data 3.** Cell-type-level differential expression analysis results. Transcript abundance and fold-changes for each gene in each cell type can also be plotted online (https://jingxianchen.shinyapps.io/l1_sc/). Related to Fig. 2.

**Sup. Data 4.** Tissue-level differential expression analysis results. Transcript abundance and fold-changes for each gene in each tissue can also be plotted online (https://jingxianchen.shinyapps.io/l1_sc/). Related to Fig. 4.

bioRxiv preprint doi: https://doi.org/10.64898/2026.09.04.749554; this version posted September 9, 2026. The copyright holder for this preprint (which was not certified by peer review) is the author/funder, who has granted bioRxiv a license to display the preprint in perpetuity. It is made available under a CC-BY 4.0 International license.



**Sup. Data 5.** Gene Ontology (GO) term analysis results. Related to Fig. 6.

**Sup. Data 6.** Results from estimation of transcription factor activities using *Cel*EsT.

**Sup. Data 7.** KEGG pathway enrichment analysis results for germline.

**Sup. Data 8.** Differential expression analysis results using intronic reads for intestine and germline.
